# Utilization of malaria prevention methods by pregnant women in Yaounde

**DOI:** 10.11604/pamj.2013.15.89.2324

**Published:** 2013-07-09

**Authors:** Calvin Ebai Bisong, Clemence Meli Dongmo

**Affiliations:** 1 Training School for Nurses and Health Technicians Yaounde, Cameroon; 2 Department of Biochemistry, Faculty of Science, University of Yaounde I, Yaounde, Cameroon

**Keywords:** Malaria, prevention methods, pregnant women, chemical methods, physical methods

## Abstract

**Introduction:**

Malaria prevention methods are diverse. Their availability sometimes does not guarantee effective usage and the use of each method in isolation may not provide the necessary results for the fight against malaria. Pregnant women are relatively more vulnerable and so it is recommended that they should be protected against malaria. Proper protection will require malaria prevention methods in combination. This study seeks to find out what methods pregnant women use and how many of them use these methods.

**Methods:**

Information on the use of malaria prevention methods was collected from pregnant women attending prenatal clinics in health institutions within the Biyem Assi health district of Yaounde VI subdivision using a pretested questionnaire. Analysis was done using SPSS version 16 (Chicago IL USA).

**Results:**

The study revealed that 82.5% of women used at least one method of malaria prevention; 12% used four methods (insecticides, bednets, indoor residual spraying and Sulphadoxine Pyrimethamine) in combination. The most used method was mosquito bednet, 82.5%. Some of the women 17.5% did not use any of the prevention methods.

**Conclusion:**

Use of malaria prevention methods in combination is not considered a priority by pregnant women. Sensitization campaigns by governments and NGOs should give that a priority position.

## Introduction

Malaria remains a great human scourge. Pregnant women and children below 5 are among the most vulnerable groups. Considering the closeness between mother and child, effective measures put in place to protect the mother from malaria could also protect the child and hence reduce the morbidity and mortality related to malaria. The World Health Organization during its Global ministerial conference on malaria in 1992 in Amsterdam, approved a number of control measures which include early diagnosis and prompt effective treatment, chemoprophylaxis in susceptible groups, reduction of man vector contact, Information Education and Communication, surveillance and research. [1, 2]. The use of these measures in pregnancy may not warrant independent programming but if used in combination to provide a range of prenatal services the incremental cost may be cost effective [3]. In view of the fact that each of these methods has its advantages and disadvantages, their use in isolation may not provide the necessary results in the fight against malaria. A randomized control trial in Gambia showed that, intermittent treated bed nets reduced overall child mortality by 50% [4]. In spite this, it is essential that mothers and would be mothers have necessary knowledge about the use and treatment of the nets, have the authorized pyrethroid insecticides at their disposal when in need and should be highly aware of the importance of the use of other malaria prevention methods [5]. Another study in Betul, central India showed that post intervention survey after indoor residual spraying (IRS) with pyrethroid insecticide showed sharp decline in the number of malaria cases and Anopheles species after an entomological monitoring [6]. The use of prevention measures in combination is a rare practice and needs attention. It could require the mobilization of huge resources but could be a possible way out in the fight against malaria in the long run. The use of mosquito bed nets has become wider than before as the distribution has known several campaigns in many countries. However not every body is in possession and not every one may be able to use it appropriately. Other measures of prevention notably IRS, use of coils and other insecticides and Sulphadoxine Pyrimethamine (SP) by pregnant women are faced with certain difficulties. In a study (The Gambia), it was found that vector control could compliment chemoprophylaxis in the prevention of malaria in pregnant women [7, 8]. SP is sometimes out of stock in health institutions; in some the distribution is inexistent. IRS is not very much known by local populations. Use of coils requires constant availability of funds and the coils. In a low income country like Cameroon it may not be very evident. The allocation of attention and funding to all prevention methods will be advantageous. In Cameroon the public health ministry has been making efforts to reduce morbidity and mortality linked to malaria. One of those measures that have received a lot of attention is the use of mosquito bednets. The national program for the fight against malaria in 2007, elaborated strategies which include: IRS, and the use of chemoprophylaxis notably SP by pregnant women [9, 10]. In conjunction with other partners the health ministry launched a campaign for free distribution of SP and insecticide treated bednets to pregnant women and chidren 0-5. Recently (October 2011), another campaign for the free distribution of long lasting insecticide treated bednets to all was launched. The objective of this study is to determine the malaria prevention methods (in isolation or in combination) used by pregnant women and how many of them use these methods.

## Methods

### Study area

This study was carried out in clinics and district hospital within the Biyem Assi health district. The period of study was between February and March 2012. The Biyem assi health district is found in the Yaoundé VI subdivision of the centre region of Cameroon. It is mainly urban and quite cosmopolitan, consisting of people from all socioeconomic status though the majority is poor. The Yaounde topography is characterized by hills and valleys and so most of the houses in the valleys usually have stagnant ponds of water, bushes and farmland surrounding them. The houses here have their walls made of either plank, mud or cement bricks. A majority of the plank and mud houses have holes on their walls creating passages for mosquitoes and other vectors to get into the houses. Yaounde is a forest zone with temperatures varying between 14-20° C in the rainy season and 26- 31° C in the dry season. There are two rainy seasons: one from August to November and the other from March to June; and two dry seasons: from December to March and June to August. The annual rainfall is about 1600mm [11].

### Study design and subjects

This was a cross sectional study involving 200 randomly selected pregnant women who came for prenatal consultations in health institutions within the Biyem assi health district. A questionnaire was given to each selected pregnant woman. This was either to be self administered or to be filled by a trained interviewer for those who could neither read nor write. The questionnaire was designed to get information on: socio-demographic factors, utilization of chemical methods of prevention and utilization of physical methods of prevention of malaria.

### Ethical approval

Administrative and ethical clearances were obtained from the divisional officer of Yaounde VI, the district medical officer for Biyem Assi health district and the head of each health institution where pregnant women were interviewed. Consent forms were issued and in some cases read out and explained to the subjects. The objective and procedure of the study was explained to the participants and those who accepted to be part of it indicated by signature.

### Sample size

We had a calculated sample size of 360; at the end of the study 200 pregnant women fully took part in the study. Some refused to participate.

### Data collection

The participants were recruited on prenatal clinic days and the pre-tested questionnaire was either filled by them or by a trained interviewer. The pregnant women were approached while waiting to take turns with the midwife. Information collected was on age, matrimonial status, occupation, level of education, age of pregnancy, utilization of SP, insecticides, mosquito bed nets and IRS.

### Statistical analysis

Data was analyzed using SPSS version 16 (Chicago IL USA). Descriptive statistics was done on variables while the Pearson chi square (X^2^) test was used to assess association between the utilization of malaria prevention methods and some social and demographic variables. Statiscal significance was cut off at p ≥ 0.05.

## Results

### Socio-demographic characteristics of the study population

A total of 200 pregnant women living within the Biyem Assi health district were involved in the study. The women were aged between 14 and 40 years with a mean (SD) age of 28 (10). A majority of the women (83%) were aged between 21 and 35 while 15.5% and 1.5% were aged less than 21 and greater than 35 years respectively. Most of them 60.5% were married, 69% had atleast attempted secondary school, 21.5% had atleast a university degree, and 8% had attempted primary school while 1.5% had no formal education. Among the women, a majority (87%) had no formal employment, they were: students (29.5%), petty traders (23.5%) and housewives (34%) while only 13% had a formal employment.

### Use of malaria prevention methods

Generally, a majority of the pregnant women (82.5%) used one or more malaria prevention methods. The most commonly used methods were insecticide treated bednets (82.5%) and SP (82.0%), 17.5% did not use any of the malaria prevention methods.

**Utilization of chemical prevention methods:** There were 82.0% (164) of the women who received SP atleast once per trimester during their prenatal consultation either from the health personnel for free or by buying from a pharmacy. Among them 79% (130) actually swallowed the tablets, 21% (34) did not. The reasons they advanced for this included: forgetfulness, dislike for tablets and the preference for traditional medicines. For those who did not receive SP, they said it was due to the lack of money to buy, not being informed and unavailability. A statistically significant association was found between the use of SP and the age group 11-15 (X^2^ = 4.572), university education (X^2^ = 4.508), no formal education (X^2^ = 4.866) and 3^rd^ trimester of pregnancy (X^2^ = 4.31). Some of the women (30%) used insecticides: mosquito coils (21.5%), raid spray (3.5%) and others (5.0%). All pregnant women who used insecticides used SP (30%). We noticed a statistically significant association between use of insecticide and age group 36-40 (X^2^ = 7.105) (Table 1).

**Table 1:** Socio-demographic characteristics of the pregnant women

Characteristics	Pregnant women (%)
**Age range (years)**	
<21	15.5
21-35	83.0
>35	1.5
**Marital status**	
Married	60.5
Unmarried	39.5
Divorced/separated	0.0
Widow	0.0
**Level of Education**	
University/postgraduate	21.5
Secondary	69.0
Primary	8.0
No formal education	1.5
**Gestational age (trimesters)**	
1^st^	5.0
2^nd^	46.0
3^rd^	49.0
**Socioeconomic status (occupation)**	
Housewife	34.0
Student	29.5
Petty trader	23.5
Formal employment	13.0
others	0.00

### Utilization of physical prevention methods

**Use of mosquito bed nets:** Among the pregnant women, 85.5% (171) indicated the possession of mosquito bed nets. However, 17.5% (30) of them declared the non use of the nets in their possession, so 82.5% (141) actually put the nets in use. Among the reasons for the non use were the following: high temperatures, feeling of being in prison, too tired to put up the net, will use after delivery and the inability of the nets to kill mosquitoes. A number of them 14.4% (29) did not have any mosquito nets. The reasons they advanced for that include: lack of money to buy, never received during the distribution by public health campaigns and the dislike for mosquito nets.

**Use of indoor residual spraying:** Nearly all the pregnant women 94% (188) indicated the non use of indoor residual spraying (IRS) as a method of malaria prevention. The 6% (12) that used IRS did so once a year using recommended pyrethroid insecticides. The reasons presented for the non use of IRS among others include: lack of knowledge on IRS (42%), fear of toxicity (17%), preference for other insecticides like coils (11%) and window nets (1%), high cost (11%), preference for mosquito nets (9%) and absence of many mosquitoes. We noticed a statically significant association between IRS and age groups 16-20 (X^2^ = 12.264) and 36-40 (X^2^ = 4.032).

**Use of both mosquito bed nets and indoor residual spraying:** all the pregnant women who used IRS also used mosquito bednets (6%) (Table 2).

**Table 2:** Utilization of chemical prevention methods

Prevention method	Pregnant women (%)
Sulpadoxine pyrimethamine	79.0
**Insecticides**	
Mosquito coils	21.5
Raid spray	3.5
others	5.0

**Use of physical and chemical methods in combination:** it was noted that the most used combination of chemical and physical methods was SP + mosquito bednets (82.09%). A minority of the women (6.0%) used the four methods (Table 3).

**Table 3:** Utilization of prevention methods singly and in combination

Prevention method	Frequency	%
**Chemical**		
SP	158	79
Insecticide	60	30
SP + insecticide	60	30
**Physical**		
Mosquito bednets	165	82.5
IRS	12	6.0
Bednet + IRS	12	6.0
**Chemical and Physical**		
SP+ bednets	164	82.0
SP+ IRS	12	6.0
Insecticide + IRS	12	6.0
Insecticide + bednets	60	30.0
Insecticide + bednet + IRS +SP	12	6.0

## Discussion

This study shows that only 6% of pregnant women use all the four methods in combination to prevent malaria, this could be due to the cost that is associated with the use of all measures in combination, considering that a majority of the population has no formal employment. SP was the most used chemical method (79%) though some women did not comply with it. This is similar to the findings of Le Port et al (2011) who found that 84% of women showed compliance to SP, with some failures in adherence recorded [12]. 82.5% of the women used atleast one or more methods of prevention. Though considerable, it is lower than 95% found by Tongo et al (2011) on the utilization of malaria preventive measures during pregnancy and birth outcomes in Ibadan, Nigeria [13].

The most commonly used combination of prevention methods was SP + bednets (82%); this could be due to the free distribution of SP by some health institutions and mosquito bednets by the government. We also noticed that not all pregnant women received SP which is supposed to be distributed free of charge to pregnant women during prenatal consultations, an indication that the drug is not readily available in some health institutions. Among the women 30% used insecticides with mosquito coils being the most used (21.5%); 30% used insecticide + bednets. These can be compared to results obtained by Ziba et al (1994) who found that among Malawian households, mosquito coils were the most used insecticides and that 47% used insecticides in combination with bednets [14]. However the greater use of insecticides in combination in our study may be linked to the campaign on free distribution of mosquito nets. Though IRS in combination with other measures has been proven to be effective to curb malaria, only 6% of women used it [6]. This could be due to its high cost and lack of knowledge on its effectiveness. The statiscally significant association between higher level of education and the use of SP (X2 = 4.508) is similar to the findings of Ebako et al (2009) who found that pregnant women with such level of education use more chemoprophylaxis than those with lower level of education (OR = 3.14; p = 0.02) [15]. This could be because with such level women can better understand the necessity for SP.

We found that third trimester pregnancy age has a statistically significant association with the use of SP (X^2^ = 4.31); this could be explained by the fact that the women at this stage must have received sensitization during their previous consultations. Statiscally significant association was also found between age range 36-40 and the use of insecticides (X^2^ = 7.205).

## Conclusion

This study shows that SP and bednets are the most used methods of malaria prevention by pregnant women either singly or in combination. Considering the importance of all methods in the reduction of morbidity and mortality linked to malaria, pregnant women should constantly be sensitized on the use of these methods especially in combination, the distribution of bednets should be evaluated and catch up organized where necessary, the distribution of SP to pregnant women should be emphasized and measures should be taken by the state and NGOs to reduce the cost of IRS.
